# Interatrial Conduction Pacing: All Roads May Lead to Rome, But Which One Should We Take?

**DOI:** 10.1016/j.jaccas.2026.107872

**Published:** 2026-04-29

**Authors:** Justin T. Tretter, Óscar Cano

**Affiliations:** a Department of Pediatric Cardiology, Cleveland Clinic Children's, and The Heart, Vascular, and Thoracic Institute, Cleveland Clinic, Cleveland, Ohio, USA; b Hospital Universitari i Politècnic La Fe, Valencia, Spain; c Instituto de Investigaciόn Sanitaria La Fe, Valencia, Spain; d Centro de Investigaciones Biomédicas en RED en Enfermadades Cardiovasculares (CIBERCV), Madrid, Spain

**Keywords:** computed tomography, electrocardiogram, electrophysiology, supraventricular arrhythmias

**Figure:**
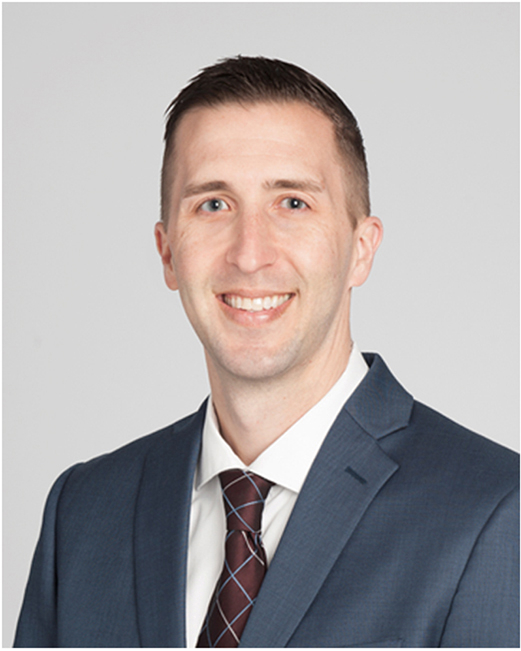

Over the past century, histological studies have established that, unlike what is found within the ventricular myocardium, there are no specialized conduction tracts within the atrial body. Instead, the electrical impulse generated by the sinus node disperses across the atrial myocardium and toward the atrioventricular node, primarily following preferential pathways of parallel aligned aggregated working cardiomyocytes.^1^ In 1916, Bachmann first described causing interatrial conduction delay by impinging upon a muscular bundle bridging the anterosuperior interatrial fold and coursing along the subepicardial anterosuperior surface of the atriums between the atrial appendages.^2^ This interatrial bundle now bearing his name has subsequently been established as the most common pathway for interatrial conduction. As there is increased attention toward the potential benefits of improved and directed cardiac pacing, it has become understood that alternative interatrial conduction pathways are not uncommon, especially in the setting of atrial myocardial disease.^3^

In the “Da Vinci Anatomy Corner” for this issue of *JACC:* *Case Reports*, González-Casal et al^4^ have provided an excellent anatomical overview of the clinical anatomy of Bachmann's bundle with discussion related to interatrial block. In describing the clinical applications, they emphasize that the structural substrate to provide interatrial conduction depends on both the myocardial architecture and vascular supply, with chronic ischemia and fibrotic remodeling serving as the main pathological mechanism for disrupting normal interatrial conduction.^4^ High-density 3-dimensional activation mapping has demonstrated marked variability in sinus node exit and subsequent interatrial propagation in adults during sinus rhythm undergoing ablation for atrial fibrillation. Specifically, sinus node exit and terminal crest interface conduction with subsequent right atrial activation wavefront serve as the key factors in determining the highly variable interatrial conduction patterns. In a mapping study, over half of the patients demonstrated interatrial conduction outside of Bachmann's bundle, namely posteriorly.^3^ While previously thought to be facilitated by small and variable muscular strands often seen traversing Waterston's groove, we believe it is more likely across the consistent and prominent musculature of the interatrial folds. Myocardial disease of Bachmann's bundle and resulting interatrial conduction block becomes even more common in those with various forms of congenital heart disease and may occur at younger ages related to the hemodynamic derangements experienced from the outset.^5^ So overall, those affected by interatrial block may represent a relatively large, and currently inadequately served, patient population.

As beautifully illustrated by González-Casal et al,^4^ understanding of the nuanced anatomy related to interatrial conduction may better guide the electrophysiologist aiming to improve or restore interatrial conduction with conduction pacing. In the clinical experience reported to date, Bachmann's bundle pacing has largely been pursued through a relatively constrained anatomical target—typically at the confluence of the superior caval vein and the so-called high interatrial septum, or superior interatrial fold. Evidence of capture has been inferred from shortening and morphological modification of the P-wave, together with the recording of discrete Bachmann's bundle potentials. Yet, appreciation of the full anatomical extent and variability of this interatrial tract suggests that the spectrum of viable implantation targets may be considerably broader, particularly when elevated capture thresholds or prolonged stimulus-to-atrial activation latency—frequently observed with excessively superior lead positioning—raise concern for suboptimal engagement of the bundle. With advances in cardiac computed tomography, this detail can now be delineated to guide procedural planning (Figure 1).^6^ This approach may potentially serve as an anatomical adjunct to intraprocedural electrical mapping, recognizing that anatomical visualization alone may not fully predict conduction. However, identifying the precaval band, a medial continuation of the terminal crest coursing anterior to the junction between the superior caval vein and right atrium, and its insertion along the anterosuperior interatrial fold directs the electrophysiologist toward the right atrial extension of Bachmann's bundle. Identification of the C-shaped interatrial folds, or limbus, can be used to identify potential alternative interatrial conduction pathways, with its superior (Figure 1B, green shading), posterior (white shading), and inferior (purple shading) segments potentially providing the described alternative interatrial pathways. Understanding and visualization of these important muscular structures may streamline intraprocedural mapping to identify the best pathway to improve or restore interatrial conduction during atrial pacing procedures.

**Figure 1:**
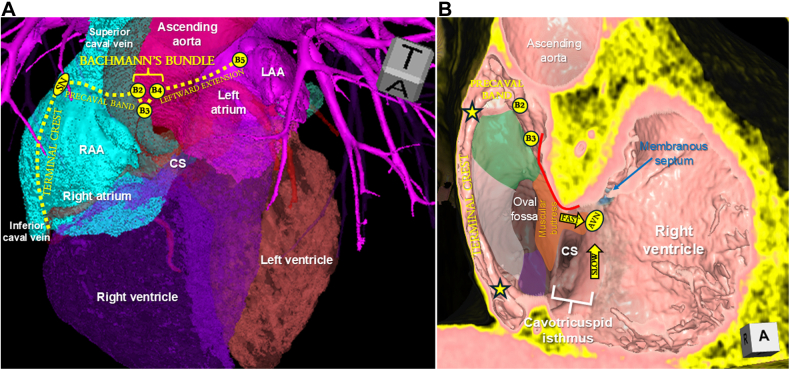
3-Dimensional Cardiac Computed Tomographic Reconstructions (A) The blood-filled cavities of the heart are visualized viewing anteriorly. The terminal crest and its precaval band (SN-B3) are marked with the yellow dotted line, and its continuation as Bachmann's bundle (B2-B4) and its leftward extension (B4-B5). (B) Virtual dissection in a right anterior oblique plane in the same heart visualizes the rightward aspect of the atrial septum. The terminal crest (labeled and with yellow stars with black borders) and its precaval band are again viewed, with the precaval band inserting at the anterosuperior interatrial fold, immediately posterior to the aortic mound (red line) formed by the noncoronary aortic sinus. The C-shaped interatrial fold is divided into superior (green shaded), posterior (white shaded) and inferior (purple shaded) segments. The muscular buttress is positioned anterior to the oval fossa and provides the fast pathway into the atrioventricular node. The cavotricuspid isthmus provides the slow pathway into the atrioventricular node. AVN = atrioventricular node; CS = coronary sinus; LAA = left atrial appendage; RAA = right atrial appendage; SN = sinus node.

## Funding Support and Author Disclosures

Dr Tretter is a consultant for Cara Medical Ltd. Dr Cano has reported no relationships relevant to the contents of this paper to disclose.
